# Circadian Rhythms of Oxidative Stress Markers and Melatonin Metabolite in Patients with Xeroderma Pigmentosum Group A

**DOI:** 10.1155/2016/5741517

**Published:** 2016-04-26

**Authors:** Rie Miyata, Naoyuki Tanuma, Hiroshi Sakuma, Masaharu Hayashi

**Affiliations:** ^1^Department of Brain Development and Neural Regeneration, Tokyo Metropolitan Institute of Medical Science, Tokyo 156-8506, Japan; ^2^Department of Pediatrics, Tokyo-Kita Medical Center, Tokyo 171-0053, Japan; ^3^Department of Pediatrics, Tokyo Metropolitan Fuchu Medical Center for the Disabled, Tokyo 183-8553, Japan

## Abstract

Xeroderma pigmentosum group A (XPA) is a genetic disorder in DNA nucleotide excision repair (NER) with severe neurological disorders, in which oxidative stress and disturbed melatonin metabolism may be involved. Herein we confirmed the diurnal variation of melatonin metabolites, oxidative stress markers, and antioxidant power in urine of patients with XPA and age-matched controls, using enzyme-linked immunosorbent assay (ELISA). The peak of 6-sulfatoxymelatonin, a metabolite of melatonin, was seen at 6:00 in both the XPA patients and controls, though the peak value is lower, specifically in the younger age group of XPA patients. The older XPA patients demonstrated an increase in the urinary levels of 8-hydroxy-2′-deoxyguanosine and hexanoyl-lysine, a marker of oxidative DNA damage and lipid peroxidation, having a robust peak at 6:00 and 18:00, respectively. In addition, the urinary level of total antioxidant power was decreased in the older XPA patients. Recently, it is speculated that oxidative stress and antioxidant properties may have a diurnal variation, and the circadian rhythm is likely to influence the NER itself. We believe that the administration of melatonin has the possibility of ameliorating the augmented oxidative stress in neurodegeneration, especially in the older XPA patients, modulating the melatonin metabolism and the circadian rhythm.

## 1. Introduction

Xeroderma pigmentosum (XP), a genetic disorder in DNA nucleotide excision repair (NER), is characterized by skin hypersensitivity to sunlight and progressive neurological impairment [1]. There are eight complementation subgroups of XP, and group A (XPA) is common in Japan, showing severe neurological disorders such as mental deterioration, cerebellar ataxia, extrapyramidal abnormalities, and neuronal deafness, but no effective treatment has been developed for neurological disorders [2]. Oxidative stress originates from an imbalance between the production of reactive oxygen species and reactive nitrogen species and the antioxidant capacities of cells and organs [3]. Oxidative stress has been confirmed to play a role in adult-onset neurodegenerative diseases, such as Alzheimer's disease [4], and we confirmed the involvement of oxidative neuronal damage in child-onset and adult neurodegenerative diseases, such as dentatorubral-pallidoluysian atrophy and superficial siderosis [5, 6]. We clarified the accumulation of oxidative stress markers in the basal ganglia in autopsy rains in XPA and Cockayne syndrome (CS), having a genetic defect in transcription-coupled repair, which results in multiple organ impairment and various neurological disorders [7]. In addition, we reported an increase in the urinary levels of oxidative stress markers in both XPA and CS in the preliminary analysis [8].

Melatonin is a functionally pleiotropic and neuroendocrine molecule and is produced mainly by the pineal gland under the control of the suprachiasmatic nucleus [9]. Melatonin regulates circadian rhythm and plays a role in the transduction of the chronobiological actions of multiple hormones. In addition, it also has antioxidant properties and anti-inflammatory abilities and is likely to be one of therapeutic tools for neurological disorders, such as multiple sclerosis and Huntington's disease [10]. Melatonin secretion has a 24-hour rhythm, in which the peak is during the midnight [11]. We preliminarily examined the urinary level of melatonin metabolite in XPA and CS, which was reduced predominantly in CS patients, suffering from the disturbed circadian rhythms of sleep-wakefulness and body temperature regulation [12].

Recently, redox regulation and/or oxidative stress has been reported to have diurnal variation, and the circadian rhythms of oxidative stress markers and antioxidant enzymes have been examined in healthy subjects and patients with neurological disorders [13]. Nevertheless, the relationships in diurnal variation between melatonin and oxidative stress markers still remain to be investigated. Herein, we analyzed the circadian rhythms of oxidative stress markers and melatonin metabolites in urine of patients with XPA.

## 2. Materials and Methods

We analyzed the urine from 8 patients with genetically confirmed XPA and 8 normal controls, aged from 6 to 37 years. It is well known that melatonin secretion varies by age. The levels of plasma melatonin and its urinary metabolites are high in infancy and early children, decline dramatically around adolescence, and keep reducing gradually in adults and aged people [14].

Accordingly, we divided both XPA patients and controls into younger and older age groups, consisting of three subjects each less than 15 years and five ones each equal to and more than 15 years, respectively. Urine samples were collected four times a day (at 0:00, 06:00, 12:00, and 18:00). All specimens were stored at −80°C in a deep freezer avoiding lights. Consents were granted from all parents of patients and child controls and adult controls. This analysis was approved by the Ethical Committee of Tokyo Metropolitan Institute of Medical Science. We measured the urinary concentrations of 6-sulfatoxymelatonin (6-SM), a main metabolite of melatonin, a marker of oxidative DNA damage, 8-hydroxy-2′-deoxyguanosine (8-OHdG), an early stage marker of lipid peroxidation, hexanoyl-lysine adduct (HEL), and total antioxidant power (TAO), respectively, as previously reported [8, 12]. We used commercially available enzyme-linked immunosorbent assay (ELISA) kits for 6-SM (GenWay Biotech, CA, USA), 8-OHdG and HEL (Japan Institute for the Aging, Shizuoka, Japan), and TAO (Oxford Biomedical Research, MI, USA), respectively.

The results were normalized to urine concentration of creatinine (Cre), except TAO. Correlations among 6-SM, 8-OHdG, HEL, and TAO at each time point were confirmed by Spearman's rank correlation coefficient.

## 3. Results

**Figure 1:**
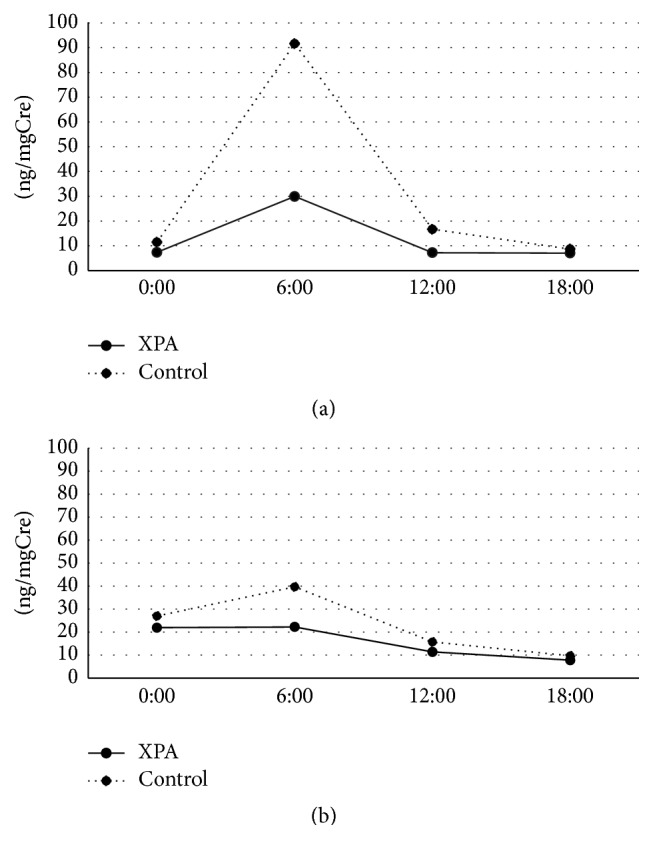
Circadian rhythm of urinary levels of 6-sulfatoxymelatonin (6-SM) (ng/mgCre) in younger age group (a) and older age group (b). Solid and dotted lines denote values of patients with xeroderma pigmentosum group A (XPA) and controls, respectively.

**Figure 2:**
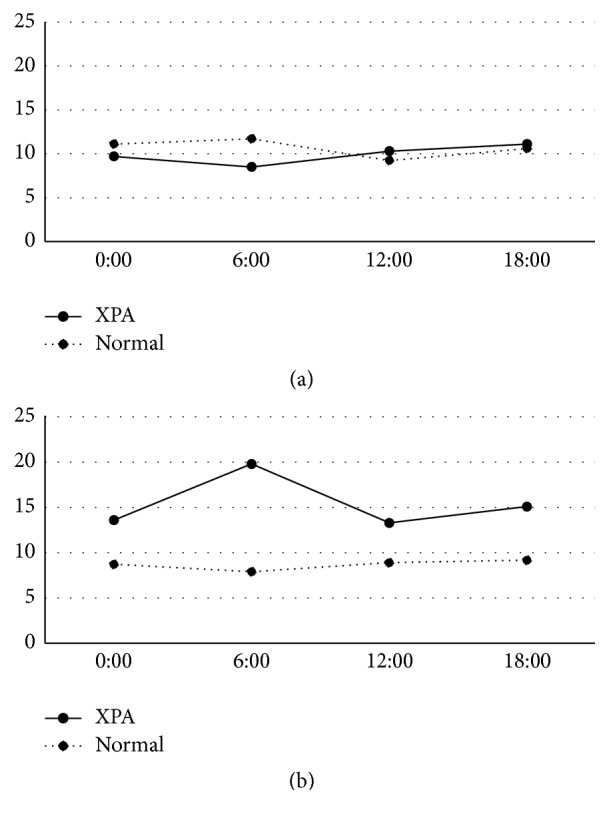
Circadian rhythm of urinary levels of 8-hydroxy-2′-deoxyguanosine (8-OHdG) (ng/mgCre) in younger age group (a) and older age group (b). Solid and dotted lines denote values of patients with xeroderma pigmentosum group A (XPA) and controls, respectively.

**Figure 3:**
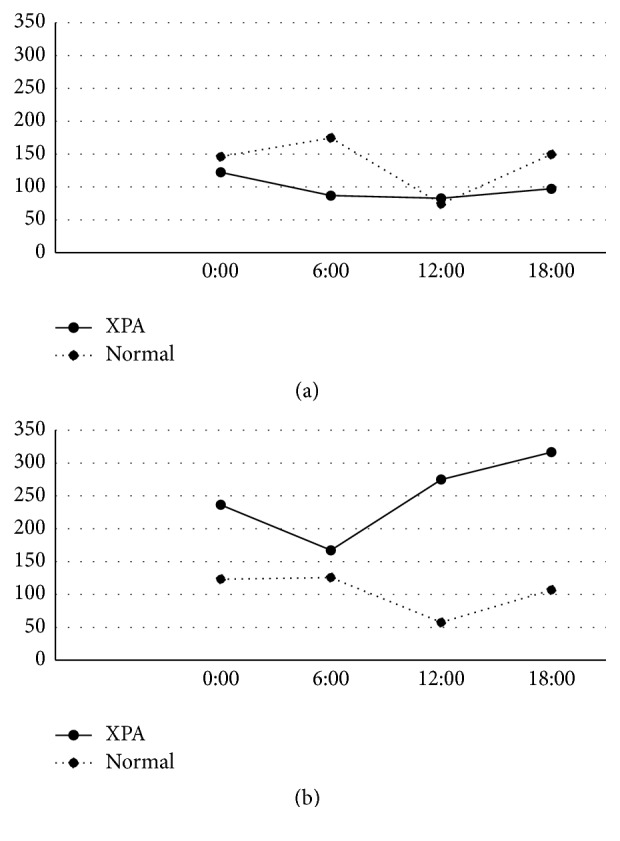
Circadian rhythm of urinary levels of hexanoyl-lysine (HEL) (pmol/mgCre) in younger age group (a) and older age group (b). Solid and dotted lines denote values of patients with xeroderma pigmentosum group A (XPA) and controls, respectively.

**Figure 4:**
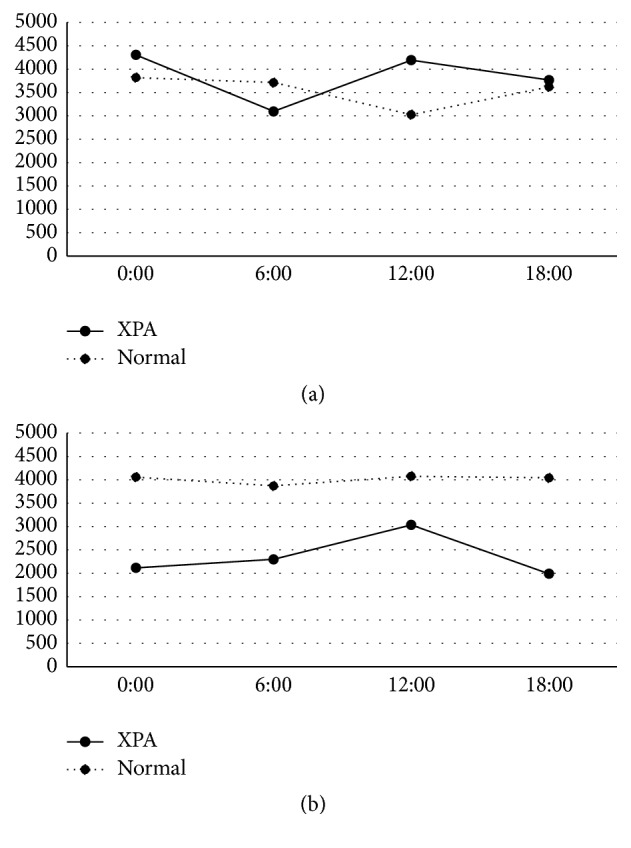
Circadian rhythm of urinary levels of total antioxidant power (TAO) (*μ*M) in younger age group (a) and older age group (b). Solid and dotted lines denote values of patients with xeroderma pigmentosum group A (XPA) and controls, respectively.

The peak of urinary 6-SM was identified at 6:00 in the younger and older age group of both controls and all XPA patients, and the peak value was reduced by age (Figure 1). The XPA patients demonstrated a lower peak value, which was approximately one-third and half of each of those in controls. The urinary levels of 8-OHdG were low and lacked the diurnal variation in the younger age group in both controls and XPA patients (Figure 2(a)). In the older age group, the urinary levels of 8-OHdG were increased in the XPA patients but not in controls, in which the robust peak was identified at 6:00 (Figure 2(b)). The urinary levels of HEL were low and lacked the diurnal variation in the younger age group in both controls and XPA patients (Figure 3(a)). In the older age group, the urinary levels of 8-OHdG and HEL were increased in the XPA patients but not in controls, in which the robust peak was identified at 6:00 and 18:00, respectively (Figures 2(b) and 3(b)). There was no significant difference in the urinary levels of TAO between the controls and XPA patients, lacking the diurnal variation in the younger age group (Figure 4(a)). In the older age group, the urinary levels of TAO in the XPA patients were reduced to half of those in controls, showing the dim peak at 12:00 (Figure 4(b)). The correlation among biomarkers could not be demonstrated statistically.

## 4. Discussion

Our preliminary study suggested the possible reduction in the urinary levels of 6-SM in XPA patients [12], which was confirmed in both the younger and older age groups in this analysis, and the presence of a dim peak is noteworthy (Figure 1). Generally XPA patients should avoid sun exposure in order to reduce the risk of ultraviolet hazard, and such lesser sunlight exposure may disturb the hypothalamic control in melatonin metabolism. Intriguingly, XPA patients rarely show circadian rhythm disturbances and/or sleep disorders except excessive daytime drowsiness [12]. As well as the reduced urinary excretion of 6-SM, the increase of urinary oxidative stress markers in aged XPA patients, which was suggested by our previous study in fewer subjects [8], was also confirmed in the older age group. Furthermore, the increased urinary secretions of 8-OHdG and HEL demonstrated the circadian rhythm with the robust peak (Figures 2(b) and 3(b)), in association with the reduced TAO (Figure 4(b)). As mentioned above, the XPA patients show various neurological disorders [1], and the aged ones tend to suffer from respiratory disturbance due to laryngeal dystonia, vocal cord paralysis, or sleep apnea [15–17]. Needless to say, the progression of neurodegeneration and/or respiratory failure can disturb the redox control in the whole body, but the reduction of melatonin secretion, occurring even in the younger age group (Figure 1(a)), may also precipitate the oxidative stress. Accordingly, we strongly speculate that the administration of exogenous melatonin may possibly correct its disturbed metabolism, alter or improve alertness, and/or prevent the exacerbation of oxidative stress.

Oxygen and circadian rhythmicity are essential in a myriad of physiological processes to maintain homeostasis, from blood pressure and sleep/wake cycles down to cellular signaling pathways that play critical roles in health and disease [13]. It is speculated, reviewing several papers, that the concentrations of many antioxidant enzymes, such as superoxide mutase, have a morning peak, while the occurrence of lipid peroxidation has an evening peak, like the peak at 18:00 in the urinary levels of HEL in the older age group of XPA patients. On the other hand, urinary markers of nucleic acid oxidation did not have diurnal variation in healthy subjects [18]. Similarly in this analysis, the controls in both the younger and older age groups did not show any circadian rhythms in the urinary levels of 8-OHdG, HEL, or TAO (Figures 2, 3, and 4). It is possible that the diurnal variation may be exaggerated in shift workers [19] and/or patients with neurological disorders like XPA. In addition, the circadian clock plays an important role in the determination of strengths of cellular responses to DNA damage and may subsequently influence the NER [20]. Accordingly, XPA patients have the potential of demonstrating a robust diurnal variation of biomarkers related to oxidative stress, because NER is genetically damaged, oxidative stress may be augmented in XPA [8], and melatonin metabolism was disturbed [12].

Unfortunately, long-lasting therapies have not been established for neurological disorders in the XPA patients. We tried the treatment with low-dose levodopa for laryngeal dystonia, providing the temporary improvement of dystonia and hand tremor [15]. Melatonin is remarkably functionally diverse with actions as a free radical scavenger and antioxidant, circadian rhythm regulator, anti-inflammatory, and immunoregulating molecule [9]. The diseases, having both the circadian rhythm disturbance and oxidative stress, are likely to benefit from therapeutic agents with the combination of circadian rhythm-resynchronizing properties and antioxidant actions. Clinical trials of melatonin have been performed for sleep problems in children with neurodevelopmental disorders, resulting in the partial amelioration of problems without adverse events [21, 22]. In Japan, we are also proceeding with a clinical trial of melatonin for sleep problems in autism spectrum disorders, and other clinical trials, using melatonin, are feasible. We strongly believe that the clinical trial of melatonin should be considered in XPA patients, and we are preparing the trial.
